# Community-based measles mortality surveillance in two districts of Katanga Province, Democratic Republic of Congo

**DOI:** 10.1186/1756-0500-6-537

**Published:** 2013-12-17

**Authors:** Alexandra A N’Goran, Ngoie Ilunga, Matthew E Coldiron, Rebecca F Grais, Klaudia Porten

**Affiliations:** 1 Epicentre, 8 rue Saint-Sabin, 75011 Paris, France; 2 Ministry of Public Health, Epidemiologic Surveillance Division, Lumbashi, Democratic Republic of the Congo

**Keywords:** Mortality, Measles, Population surveillance, Democratic Republic of the Congo

## Abstract

**Background:**

Mortality due to measles is often under-reported. Traditional methods of measuring mortality can be time and resource-intensive. We describe the implementation of a community-based method to monitor measles mortality.

**Findings:**

Using standardized questionnaires in the midst of a measles outbreak, a community-based network of volunteers recorded a much larger number of deaths (376) than deaths recorded in health centres (27). Deaths were predominantly (93.5%) among children aged less than 5 years; 54.5% of measles deaths reported antecedent measles vaccination.

**Conclusions:**

In this setting, the number of deaths due to measles reported in community-based surveillance was much higher than deaths reported in health centres. Lack of reliable population data and incomplete coverage of the surveillance system make it impossible to calculate overall attack rates and cause-specific mortality rates. Similar systems could be rapidly implemented in other difficult outbreak settings.

## Findings

### Background

Measuring measles mortality can be difficult, and estimates of measles case fatality ratios vary widely depending on the context [1]. Retrospective mortality surveys can be time- and resource-intensive, and not always feasible in low-income and crisis settings [2].

In 2011, a measles epidemic affected Katanga Province in south-eastern Democratic Republic of Congo (DRC), with tens of thousands of cases reported across the province. We describe a community-based measles mortality surveillance system put in place at the height of the epidemic.

### Methods

Two parallel surveillance systems were put in place in 39 non-contiguous Health Areas (HA) of the Katanga Province. Difficult access precluded the inclusion of all HAs in the zone affected by the epidemic. Firstly, a register-based system collecting clinical, demographic, and vital status information about measles cases was instituted in each of the Health Centres of the HAs included.

Secondly, a network of community volunteers (CVs) carried out active measles mortality surveillance. The CVs regularly asked community leaders to identify deaths and then collected data on measles deaths on a weekly basis from surviving heads of households using a standardized data collection tool after obtaining oral consent. Data on measles deaths were collected retrospectively for the period from April 1 to 30, 2011, and then prospectively from May 1 to 31, 2011. In the case of a death being captured by both the register-based and community-based systems, information was collected from the register, and the death was classified as being captured by the register system.

A measles case was defined as an illness characterized by fever, rash and at least one of the following symptoms: cough, coryza, or conjunctivitis [3]. A measles death was defined as a death occurring within 30 days of the onset of rash, and not due to another obvious cause, such as trauma.

Data were entered using EpiData v3.1 (EpiData Association, Odense, Denmark) and analysed using Stata v11.0 (StataCorp, College Station, USA).

The protocol for the surveillance was accepted by local, regional and national health authorities. The data presented here are the product of this surveillance system. This data collection followed the principles set forth in the Helsinki Declaration. Surveillance and field surveys are considered to be exempt from the Medecins Sans Frontieres Ethical Review Board as they represent a standard component of program monitoring. No specific ethnic or identifying information was recorded and all individuals were free to refuse contributing information to the surveillance system.

### Results

A total of 10 742 measles cases were registered in the HCs during the study period. Only 27 deaths were recorded in the HCs, whereas 376 were captured in the community surveillance system. Information about measles deaths is presented in Table 1. 93.5% of measles deaths occurred in children aged less than 5 years.

**Table 1:** Characteristics of measles deaths (N = 403), 39 health areas of Katanga Province, Democratic Republic of Congo, April-May 2011

**Characteristic**	**Number**	**Percentage**
*Place of reporting*		
Reported in community	376	93.3%
Reported in health centres	27	6.7%
Sex^*^		
Male	215	53.6%
Female	186	46.4%
Age^*^		
<1 year	107	26.7%
1-4 years	268	66.8%
5-14 years	26	6.5%
History of measles vaccination**		
Yes	214	54.5%
No	179	45.5%

^*^Age and sex missing for 2 deaths.

**Vaccine status missing for 10 deaths.

Only measles deaths, and not measles cases, were recorded at the community level. It was therefore impossible to compare case fatality ratios (CFR) observed using the two systems. However, considering all deaths observed in the two systems (403) and the 10 742 cases notified at the level of the HCs, the death-to-case ratio was 3.7%.

220 of the 403 measles deaths (54.5%) reported a prior history of measles vaccination. Time between symptom onset and death was collected in the community system, with the median time being 6 days (IQR: 4–11). These data were not collected in HCs.

### Discussion

Our results show the difficulties of relying on HC-based mortality reporting: most measles deaths during this epidemic occurred at home and were not recorded in the passive surveillance system. By implementing active, community-based measles mortality surveillance, we captured a greater number of reported measles deaths during this epidemic. The overall death-to-case ratio of 3.7% seen is in line with CFRs observed during retrospective mortality surveys of epidemics in similar contexts [1].

It is surprising that 54.5% of deaths reported a prior history of measles vaccination, but it should be noted that this was not confirmed by vaccination cards. For vaccinations administered in the distant past, it is possible that recall bias may have affected these results – with surviving parents potentially misclassifying doses of different vaccines. In the midst of an epidemic, it is also possible that some measles cases and deaths occurred in the days immediately following measles vaccination, before immunity was able to develop.

While not as precise as a retrospective mortality survey, the mortality surveillance system nonetheless provided a realistic estimation of measles mortality in this setting, while using minimal time and resources.

A recall bias could have affected the classification of measles deaths reported retrospectively, but this is unlikely given the short recall period. The inability to calculate true CFRs is a limitation; however, the large difference between measles deaths reported in the community and in HCs reaffirms the overall impression that reliance on HC-based mortality surveillance leads to under-reporting of measles deaths.

Similar community mortality surveillance systems could be implemented in future measles outbreaks, particularly when logistic concerns preclude the possibility of performing retrospective mortality surveys. The partial prospective nature of the system would also allow for reactive interventions when problem areas are identified.

## Competing interests

The authors declare that they have no competing interests.

## Authors’ contributions

AN designed and implemented the surveillance system, and was responsible for manuscript drafting. NI helped to implement the surveillance system in DRC. RG, MC and KP provided substantial contributions to the design of the surveillance system and to the revision of the manuscript. All authors read and approved the final manuscript.

## References

[B1] Wolfson LJ Grais RF Luquero FJ Birmingham ME Strebel PM Estimates of measles case fatality ratios: a comprehensive review of community-based studies Int J Epidemiol 2009 6 192 205 10.1093/ije/dyn224 19188207

[B2] Checchi F Robert L Documenting mortality in crises: what keeps us from doing better? PloS Med 2008 6 7 e146 10.1371/journal.pmed.0050146 18597552 PMC2443202

[B3] Cairns K Nandy R Grais R Challenges in measuring measles case fatality ratios in settings without vital registration Emerg Themes Epidemiol 2010 6 4 10.1186/1742-7622-7-4 20642812 PMC2918600

